# Uncertainties in Pollution and Risk Assessments of Heavy Metals in Lake Sediments Using Regional Background Soils in China

**DOI:** 10.3390/toxics11070613

**Published:** 2023-07-14

**Authors:** Dejun Wan, Jiapeng Gao, Ruiting Song, Lei Song, Dongliang Ning

**Affiliations:** 1 School of Geographical Science, Nantong University, Nantong 226007, China; gaojiapeng1002@foxmail.com (J.G.); ruitingsong@foxmail.com (R.S.); ningdl@ntu.edu.cn (D.N.); 2 Institute of Hydrogeology and Environmental Geology, Chinese Academy of Geological Sciences, Shijiazhuang 050061, China; sdsonglei@163.com

**Keywords:** sediment pollution, toxic elements, quality criteria, contamination assessment, background determination, water pollution, China

## Abstract

Background soils are frequently utilized as a surrogate to assess pollution levels and environmental risks of heavy metals in Chinese lakes. However, there remains a lack of understanding regarding the reliability and uncertainty of such assessments. Here, we determined heavy metals (As, Cd, Co, Cr, Cu, Hg, Ni, Pb, and Zn) in sediment cores from five rural lakes in North China to evaluate the reliability and uncertainty of the assessments using background soils by comparing them with assessments based on background sediments. Comparative studies reveal large uncertainties in the assessments using background soils. Among these metals, uncertainties for Hg and Cd are relatively large, whereas those for the other metals are minor. This discrepancy is due to the considerably higher natural variability of Hg and Cd in soils and sediments in comparison to the other metals. Generally, assessments utilizing background soils underestimate pollution levels and risks of Hg but overestimate those of Cd in these lakes. Despite limited human activities around the lakes, they still received a considerable influx of heavy metals via regional atmospheric transport. Assessments of the nine metals indicate moderate to considerable ecological risks in these lakes. The risks are contributed primarily (78–89%) by Hg and Cd. This study underscores the substantial uncertainties in assessing heavy metal pollution and risks using regional background soils and emphasizes the importance of controlling atmospheric emissions of Hg and Cd to mitigate pollution in rural and remote water bodies in China.

## 1. Introduction

Lakes constitute a significant portion of surface freshwater in China and play a crucial role in water supply, flood control, landscape entertainment, and the preservation of regional biodiversity [1,2,3]. However, due to rapid developments in industry and economy over the past few decades in China, an increasing number of lakes, both urban and rural, have been reported to be contaminated with various pollutants, particularly heavy metals [1,2,4,5,6,7].

Sediment is the ultimate sink of heavy metals in lakes, and thus it is widely employed as a geo-marker for assessing heavy metal pollution and environmental risks and for identifying potential pollution sources [8,9,10,11,12]. In recent years, numerous studies have been conducted to investigate heavy metals in lake sediments, aiming to determine their pollution status, ecological and health risks, as well as potential sources [1,4,5,7,13,14,15,16,17,18,19,20]. These studies have furnished valuable data for comprehending heavy metal pollution in Chinese lakes.

However, owing to the lack of geochemical baselines for heavy metals in lake sediments, most previous studies conducted heavy metal pollution and risk assessments by referring to regional background soils as a surrogate [1,4,5,7,17,19,21]. Given the differences in chemical compositions between soil and lake sediment [10] and the spatial variabilities in metal concentrations in regional soils [22], there may be varying degrees of uncertainty in such assessments. Additionally, many studies have only focused on a subset of heavy metals in lake sediments and have often not included some important toxic metals like Hg [4,6,16,20,21]. Consequently, there is an urgent need to establish reliable geochemical baselines in representative lakes in China to assess the uncertainty/reliability of pollution and risk assessments by referring to regional background soils and to conduct comprehensive pollution assessments including as many toxic metals (metalloids) as possible, such as Hg, As, Cd, and Pb.

**Figure 1:**
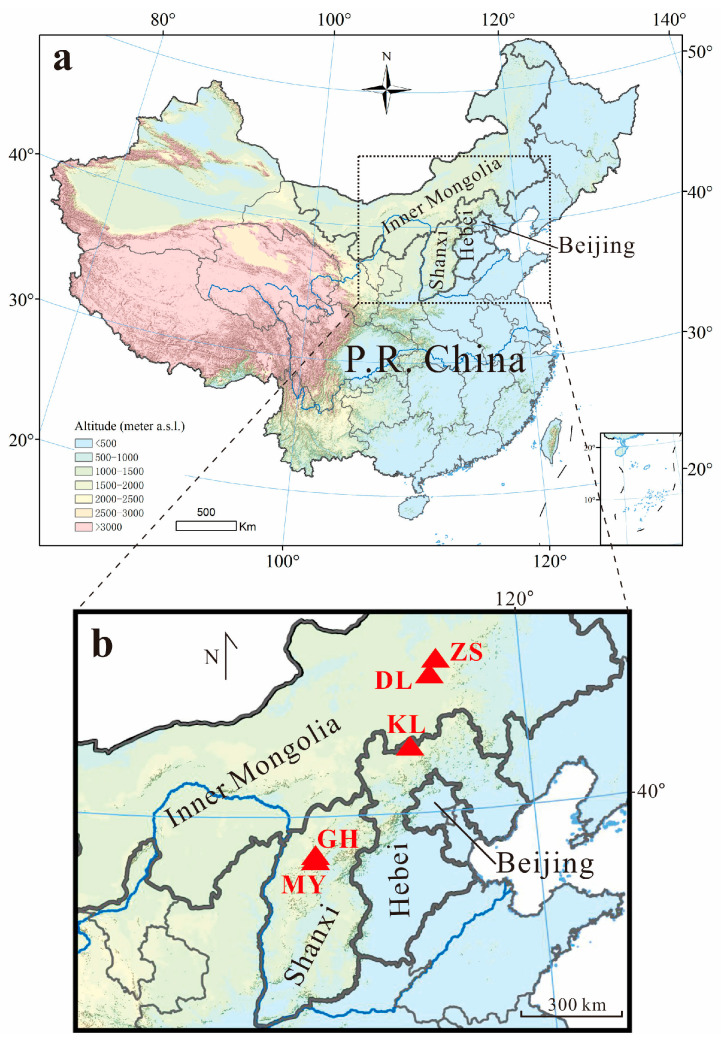
(**a**) The study area in China. (**b**) Locations of lakes Gonghai (GH) and Mayinghai (MY) in Shanxi Province, Kulunnao (KL) in Hebei Province, and Dalihu (DL) and Zhagesitai (ZS) in Inner Mongolia in North China.

Lakes situated in remote areas are frequently subjected to minimal human activity. They usually contain naturally deposited sediments with limited human disturbance. As a result, these lakes provide an ideal opportunity to examine the pre-pollution conditions of sediments and assess the reliability/uncertainty of heavy metal assessments using background soils by comparing them with assessments based on background sediments. Therefore, five representative lakes situated in relatively remote areas across three provinces in North China (Figure 1) were selected for investigation. Sediment cores were retrieved from these lakes with the aim of accomplishing the following objectives: (1) determining concentrations of nine heavy metals (As, Cd, Co, Cr, Cu, Hg, Ni, Pb, and Zn) in lake sediments; (2) establishing the background value of these metals by examining the pre-pollution conditions of sediments in each lake; (3) evaluating the uncertainties in pollution and risk assessments of heavy metals in lake sediments using regional background soils as references; and (4) assessing the status of heavy metal pollution and potential ecological risks in the sediments of these lakes. The outcomes of this study hold implications for the selection of geochemical baselines for assessing heavy metal pollution/risks in lake sediments as well as for comprehending the current status and sources of heavy metal pollution in rural lakes in China.

## 2. Materials and Methods

### 2.1. Study Area

In this study, five typical lakes, Gonghai (GH), Mayinghai (MY), Dali (DL), Zhagesitai (ZS), and Kulun (KL), from three provinces in North China were chosen for investigation (Figure 1). Generally, the lakes are situated in relatively remote or rural areas and were affected by few human activities related to heavy metal pollution surrounding the lakes. Lakes GH and MY are sub-alpine lakes. They are situated on the remote Lvliang Mountain in Xinzhou City in central Shanxi Province [23,24]. Lakes DL and ZS lie at the north margin area of the E–W trending Hunshandake Desert Land on the southeastern Mongolian Plateau [25]. Lake KL is situated in a rural area in Zhangjiakou, North Hebei. There are some villages surrounding the lake, but there are few industrial activities related to heavy metal emissions. To the south (about 5 km) of the lake is a small county (Guyuan) with a backward economy [26]. Other details about the lakes can be found in Table 1.

### 2.2. Sampling and Analysis

In Shanxi Province, two sediment cores were retrieved from lakes GH and MY in 2014 using a gravity core with a 90-millimeter-diameter polycarbonate tube. The other three were sampled from lakes DL, ZS, and KL in 2016. The sediment cores were specifically taken from the lake center areas GH, MY, ZS, and KL with water depths of about ~10 m, ~9 m, 1.8 m, and 4.0 m, respectively, and from a deep water area (approximately 10 m) in lake DL [23]. Some other details, such as lengths and locations, of the sediment cores can be found in our previous studies [23,25,26,27]. Once the sediment cores were obtained, they were sectioned at 1.0 cm intervals. Subsequently, the sediment samples were weighed, freeze-dried in a vacuum-freezing dryer at approximately −40 °C, and homogenized. Finally, a portion of each sample was ground to a powder smaller than 63 μm and stored in plastic bags for subsequent chemical analysis.

Mercury concentrations in the sediment cores from GH were pretreated and measured at the Environment Research Center in University College London, following the method described by Yang et al. (2016) [28]. Initially, the samples were digested using 8 mL of aqua regia in 50 mL of polypropylene DigiTUBE (SCP Science) on a hot plate, maintaining a temperature of 100 °C for a duration of 1.5 h. Subsequently, mercury concentrations in the digested solution were measured using cold vapor-atomic fluorescence spectrometry (CV-AFS) after undergoing reduction with SnCl_2_. Quality control was implemented by digesting and analyzing analytical blanks and standard reference materials (stream sediment GBW07305, certified Hg value 100 ± 10 ng g^−1^). The average recovery rates for Hg in the reference material GBW07305 were determined to be 93 ± 8%. For the other sediment samples, mercury concentrations were analyzed using a direct mercury analyzer (Hydra-C, Leeman Labs Inc., Hudson, NH, USA) following the USEPA method at the State Key Laboratory of Lake Sciences and Environment [26]. Standard reference material (GSD-23) and duplicate measurements were employed to ensure the quality of the analysis. The measurement errors were found to be below 5%, with a detection limit of 0.6 ng g^−1^.

To measure concentrations of various heavy metals such as Ti, Zn, Co, Cr, Ni, Cu, As, Cd, and Pb, sediment samples were first digested in Teflon tubes using a combination of hydrochloric acid, perchloric acid, hydrofluoric acid, and nitric acid at temperatures ranging from 145 to 165 °C [27,29,30]. This digestion process was carried out at the State Key Laboratory of Lake Science and Environment in China. Once fully digested, the samples were diluted to a fixed volume of 50 mL using double-distilled deionized water. Concentrations of major elements (Ti and Zn) were determined using an inductively coupled plasma atomic emission spectrometer (ICP-AES; Leeman Labs Inc., USA), while concentrations of other metals were determined using an inductively coupled plasma mass spectrometer (ICP-MS; Agilent 7700x, Santa Clara, CA, USA). To ensure the quality of the analyses, duplicate measurements and standard geological reference materials (GSD-23 and GBW07358) were employed as controls.

Furthermore, in order to establish the background (pre-pollution) and present (post-2010) sediments, the activities of ^137^Cs, ^210^Pb, and ^226^Ra in the sediment cores were detected with a low-background germanium detector. Specifically, the activities of ^210^Pb were measured at 46.5 keV, while those of ^137^Cs were at 662 keV. The activities of ^226^Ra were detected at 295 keV and 352 keV, and γ-rays were emitted by its daughter isotope, ^214^Pb [23,31]. Moreover, chronologies of the sediment cores were determined using the constant rate of ^210^Pb_ex_ supply (CRS) model in combination with ^137^Cs. The dating details and results can be found in our previous studies by Wan et al. (2020 and 2022) [23,26]. Finally, two, two, three, eight, and five fractions in the top (post-2010) sediment cores of GH, MY, DL, ZS, and KL were selected to be present sediments for study, respectively.

### 2.3. Pollution and Ecological Risk Assessments

#### 2.3.1. Enrichment Factor (EF)

The enrichment factor (EF) is a widely employed index in assessing the enrichment and pollution levels of heavy metals in soil and sediment, as well as determining their natural and anthropogenic sources [32,33,34]. The EF of a metal in sediment can be calculated using the following equation:

EF = (X_i_/X_r_)_sediment_/(X_i_/X_r_)_background_ (1)

where X_i_ and X_r_ are concentrations of metal i and the reference element (Ti) in sediment samples and in the background materials, respectively. In this study, background sediments in the five lakes and natural soils in the three provinces (Shanxi, Inner Mongolia, and Hebei) [22] were considered the background materials.

The EF values allow for the classification of heavy metal enrichments into five categories: (1) EF ≤ 2, low enrichment; (2) 2 < EF ≤ 5, moderate enrichment; (3) 5 < EF ≤ 20, high enrichment; (4) 20 < EF ≤ 40, very high enrichment; and (5) EF > 40, extremely high enrichment [33,34].

#### 2.3.2. Nemerow Pollution Index (NPI)

The Nemerow pollution index (NPI) is a weighted multi-factor index used to assess the comprehensive pollution status of all heavy metals present in sediments [35,36,37]. This index focuses on the most highly polluted metals. The calculation of NPI is outlined as follows:

NPI = [(PI_ave_)^2^ + (PI_max_)^2^]^1/2^ (2)

PI = C_i_/B_i_ (3)

In the formulas, PI represents the pollution index (PI) of metal i. PI_ave_ represents the average value of PI for all the studied heavy metals in a given sediment sample, and PI_max_ is the maximum value of PI among the heavy metals. C_i_ and B_i_ denote concentrations of metal i in the sediment sample and in the background material, respectively. It is worth noting that both the NPI and PI can be categorized as follows [35,36,37]: NPI/PI ≤ 1, unpolluted; 1 < NPI/PI ≤ 2, slightly polluted; 2 < NPI/PI ≤ 3, moderately polluted; NPI/PI > 3, highly polluted.

#### 2.3.3. Potential Ecological Risk

The calculation for determining the potential ecological risk of metal i in sediment is expressed as follows [38,39,40,41]:

(4) $$E_{i}=T_{i}\;\times \;\frac{C_{s}^{i}}{C_{b}^{i}}$$

(5) $$\mathrm{RI}=\sum E_{i}$$

where E_i_ represents the potential ecological risk of metal i in the sediment. T_i_ (Hg = 40, Cd = 30, As = 10, Co = Ni = Cu = Pb = 5, Cr = 2, and Zn = 1) denotes the toxic-response factor of metal i [38]. $C_{s}^{i}$ denotes the concentration of metal i in the sediment. $C_{b}^{i}$ denotes the concentration of metal i in the background material. The RI is the potential ecological risk of all the studied metals in the sediment.

Ecological risk levels associated with heavy metals can be categorized into the following classes [38,39,40]: E_i_ < 40, RI < 150: low ecological risk; 40 ≤ E_i_ < 80, 150 ≤ RI < 300: moderate ecological risk; 80 ≤ E_i_ < 160, 300 ≤ RI < 600: considerable ecological risk; 160 ≤ E_i_ < 320, 600 ≤ RI < 1200: high ecological risk; and E_i_ ≥ 320, RI ≥ 1200: very high ecological risk.

## 3. Results and Discussion

### 3.1. Heavy Metals in the Five Lakes’ Present and Background Sediments and in Regional Background Soils

Statistical values of heavy-metal concentrations in present and background sediments in the lakes, as well as in regional background soils, are shown in Table 2.

In Shanxi Province, the two lakes of GH and MY have roughly similar metal concentrations, except Hg, in their background sediments (Table 2), corresponding well with the fact that they are only ~5 km away from each other. For Hg, its background concentration (19.1 ± 2.2 μg kg^−1^) in GH is only half of that in MY (39.4 ± 1.8 μg kg^−1^). The large discrepancy in background Hg between the two lakes can also be found in another study by Liang et al. (2022) [42]. Metal concentrations, except Hg, in the two lakes’ background sediments are close to those in Shanxi background soil (Table 2). Mercury concentration (39.4 ± 1.8 μg kg^−1^) in MY background sediments is 1.6 times higher than the soil (24.0 ± 31.1 mg kg^−1^), whereas that in GH (19.1 ± 2.2 μg kg^−1^) is only 80% of the soil.

In the present (2010–2014) sediments, concentrations of As, Cd, Pb, and Hg in MY and of Cd, Pb, and Hg in GH are higher than their background values, while other metals are similar or slightly low (Table 2). Among the metals, the most significant differences are found in Cd (~2-fold) in MY and in Hg (3.4-fold) in GH compared with their sediment backgrounds. Although Pb is one of the most prevalent pollutants in the world, its values in the two lakes’ present sediments are only 20–30% higher relative to their background sediments.

In Inner Mongolia, the two lakes DL and ZS are ~40 km away from each other and both situated in the Hunshandake Sandy Land with similar geological environments (Figure 1). Hence, they have roughly similar metal concentrations in their background sediments (Table 2). Compared with the two lakes in Shanxi Province, the discrepancy in metal concentrations between lake background sediments and regional soils is larger in Inner Mongolia. Among the metals, Cd has the largest discrepancy. The Cd concentrations in DL and ZS background sediments are remarkably (2.3–2.9-fold) higher than those in the Inner Mongolia background soils (24.0 ± 31.1 mg kg^−1^) (Table 2). In contrast, Hg concentrations (12.5 ± 1.2 μg kg^−1^ in DL and 22.3 ± 1.4 μg kg^−1^ in ZS) in the two lakes’ background sediments are obviously lower than those in the soil (34.0 ± 46.1 μg kg^−1^).

In the present (2010–2016) sediments, concentrations of Zn, As, Cd, Pb, and Hg in DL and of Zn, Ni, Cu, Cd, Pb, and Hg in ZS are higher than their background sediments, while other metals are roughly similar. Among the metals, the Hg content in the two lakes’ present sediments is obviously (2.2–3.1-fold) higher than that in their background sediments. The second is Cd, about one-fold higher. Pb and other metals (Zn, Ni, and Cu) in the two lakes’ present sediments are slightly (20–30%) higher than those in their background sediments.

In Hebei, heavy metal contents in the background sediments of KL are close to those in DL and ZS (Table 2), as they are all situated in the southeastern Mongolian Plateau (Figure 1). Compared with Hebei background soil, most metals, including Zn, Cr, Co, Ni, Cu, Pb, and Hg, in KL background sediments are lower, whereas Cd and As are higher (Table 2). In the present (2010–2016) sediments in KL, concentrations of Zn, Cd, Pb, and Hg are higher than those in its background sediments, while other metals are roughly similar. Similar to other lakes, the most significant difference is also found in Hg. Its present value is 5.7 times the background. In addition to Hg, other metal concentrations, such as Zn, Cd, Ni, and Pb, in the present sediments are also obviously higher than those in the background.

### 3.2. A Comparison of Assessing Metal Pollution and Risks between Using Regional Background Soils and Background Sediments in These Lakes

**Figure 2:**
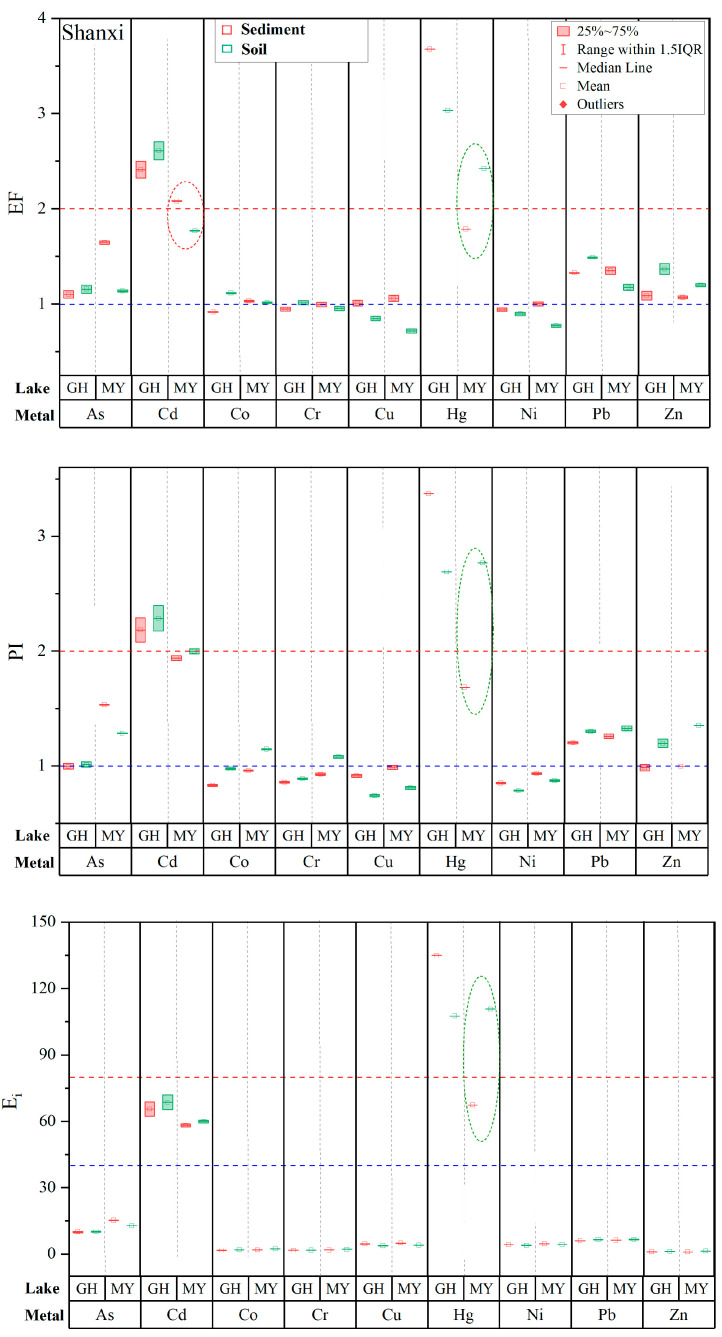
A comparison of enrichment factors (EF), pollution indexes (PI), and potential ecological risks (E_i_) of heavy metals assessed using background soils and background sediments in lakes GH and MY in Shanxi Province. Dotted blue and red lines in the third sub-figure represent E_i_ = 40 and E_i_ = 80 (also in Figure 3 and Figure 4). Dotted ellipses represent heavy metals with detectable pollution and risks and discrepancies of values between the two backgrounds higher than 50% or reaching a level (also in Figure 3 and Figure 4).

**Figure 3:**
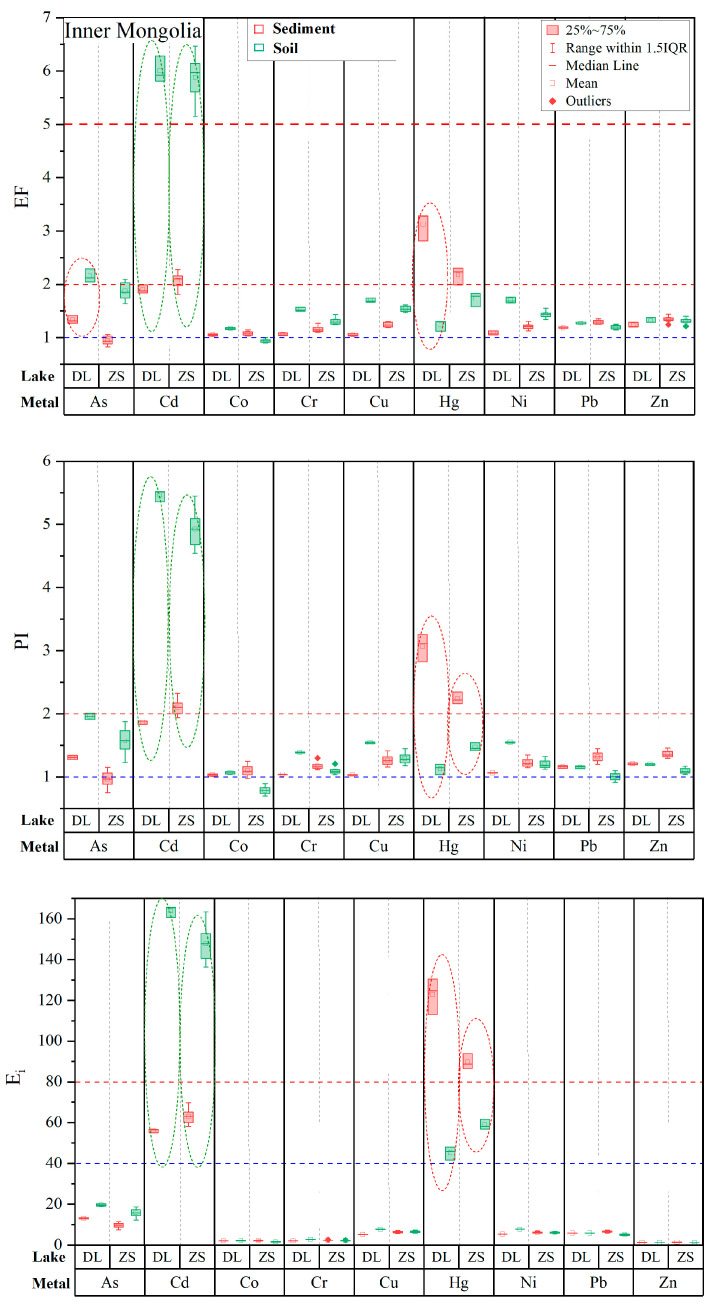
A comparison of enrichment factors (EF), pollution indexes (PI), and potential ecological risks (E_i_) of heavy metals assessed using background soils and background sediments in lakes DL and ZS in Inner Mongolia.

**Figure 4:**
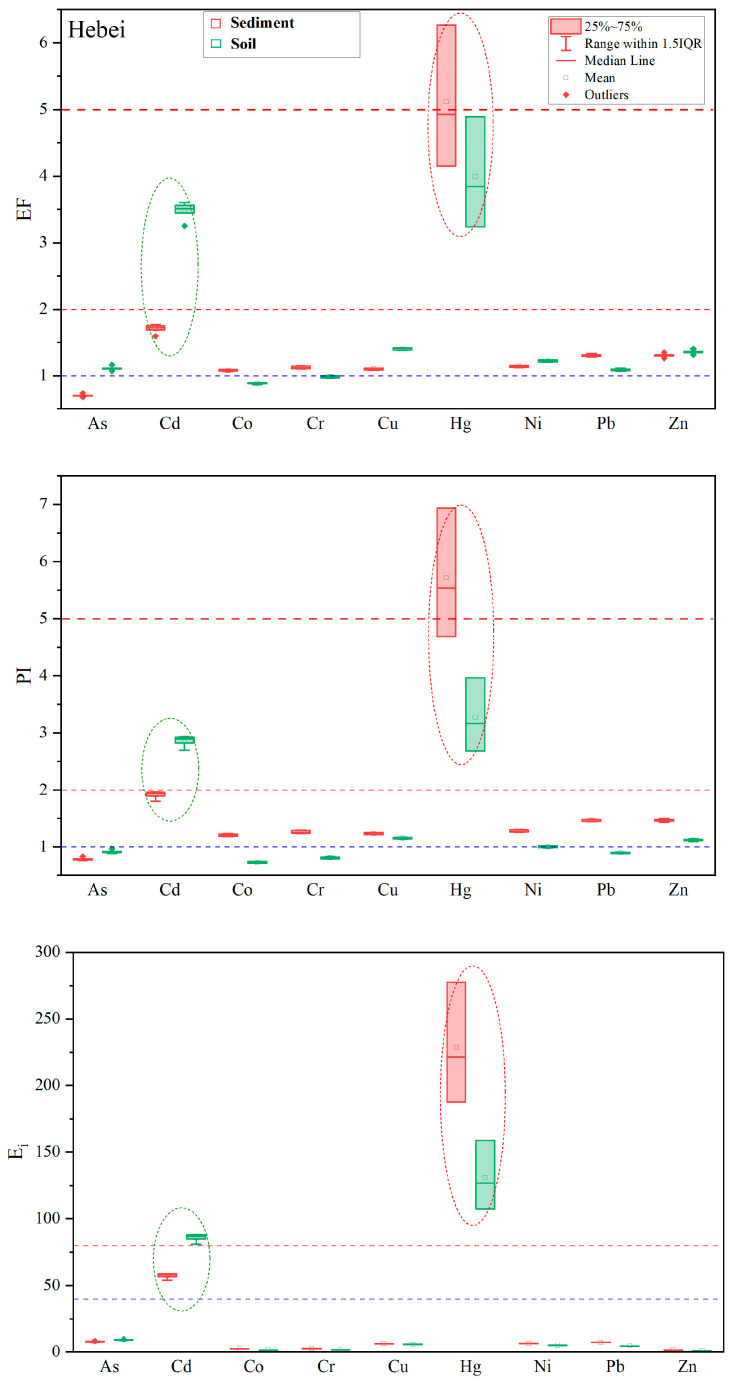
A comparison of enrichment factors (EF), pollution indexes (PI), and potential ecological risks (E_i_) of heavy metals assessed using background soils and background sediments in lake KL in Hebei Province.

To evaluate the reliability/uncertainty of pollution and risk assessments of heavy metals in lake sediments using background soils as a surrogate, in the following we compare pollution (EF and PI) and risk (E_i_) results for the nine metals in the five lakes by referring to background soils and background sediments. In Figure 2, Figure 3 and Figure 4, heavy metals with detectable pollution and risks and discrepancies of values between the two backgrounds higher than 50% or reaching a level are marked with ellipses.

In the two lakes in Shanxi Province, most metals show similar values of EF, PI, and E_i_ by comparing the two backgrounds (Figure 2). Relatively large discrepancies are only found in MY for Hg (EF, PI, and E_i_) and Cd (EF). The assessment results of Hg by referring to background soils are one level higher than those by referring to background sediments.

In the two lakes in Inner Mongolia, three metals (Cd, As, and Hg) show relatively large discrepancies between the two backgrounds (Figure 3). For Cd, its pollution and risk values based on background soil suggest highly polluted and considerable to high ecological risks, whereas those based on background sediment suggest slightly to moderately polluted and moderate ecological risks. In contrast, the pollution and risk values of Hg based on background soil suggest slightly polluted and moderate ecological risks, whereas those based on background sediment suggest moderately polluted and considerable ecological risks.

In lake KL in Hebei Province, a large discrepancy between the two backgrounds is found in Cd and Hg (Figure 4). For Cd, its pollution and risk values based on background soil suggest moderately polluted and considerable ecological risks, whereas those based on background sediment suggest slightly polluted and moderate ecological risks. In contrast, the pollution and risk values of Hg based on background soil are highly polluted and of considerable ecological risk, whereas those based on background sediment suggest very high pollution and ecological risk.

**Figure 5:**
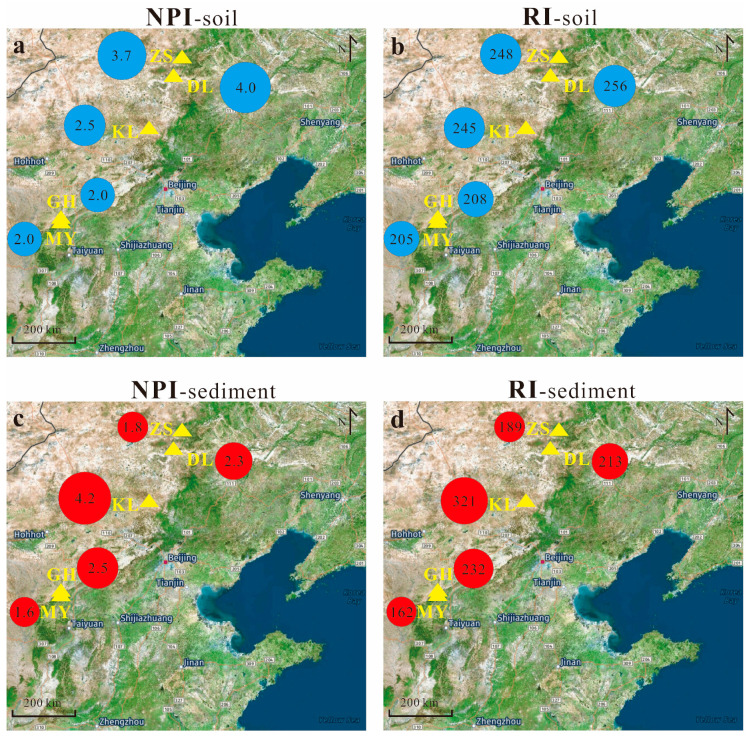
The Nemerow index (NPI) and potential ecological risks (RI) of the nine heavy metals (As, Cd, Co, Cr, Cu, Hg, Ni, Pb, and Zn) in the present sediments of the five lakes were calculated by referring to regional background soils (**a**,**b**) and background sediments (**c**,**d**).

From Figure 5, it can be seen that NPI values in lakes of ZS and DL calculated by referring to background soils are obviously higher than those calculated by referring to background sediments. In contrast, the NPI in KL based on background soils is obviously lower than that based on background sediments. In the other two lakes, GH and MY, their NPIs are relatively low, and their discrepancies in NPI between using different backgrounds are relatively small. RI values in these lakes show similar discrepancies with NPI between the two backgrounds.

From the above comparisons of pollution and risk assessment results of heavy metals in the five lakes from three provinces in North China, it can be found that there are obvious discrepancies in the assessments between the two backgrounds, implying large uncertainties in environmental assessments of heavy metals by referring to regional background soils instead of background sediments in Chinese lakes. Generally, uncertainties for Hg, Cd, and occasionally As are relatively large, whereas those for the other metals are minor. The assessments made by referring to background soils usually underestimate pollution levels and risks of Hg but overestimate those of Cd in these lakes.

### 3.3. Causes for the Uncertainties in Assessing Heavy Metal Pollution and Ecological Risks Using Regional Background Soils

**Figure 6:**
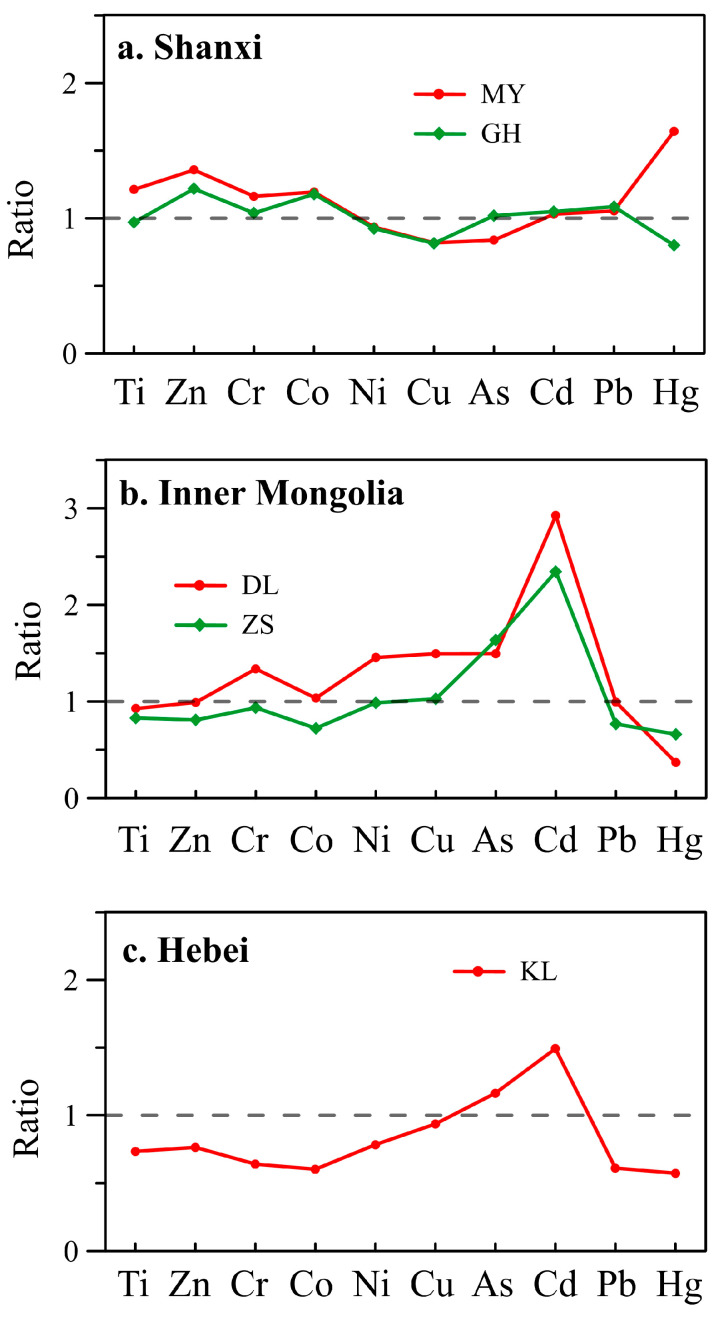
Ratios of heavy metals in the background sediments of the five lakes to those in regional background soils in the three provinces.

A direct reason for the uncertainties is the ubiquitous differences in metal concentrations between the regional background soils and the background sediments in the lakes (Figure 6). The widely used background soils in China are obtained by investigating soils from dozens to hundreds of representative sites in each province [22]. Most provinces in China cover an area of tens of thousands or even millions of square kilometers. A province often contains a variety of parent rock and soil types, resulting in great spatial heterogeneities in metal content in soils. Therefore, averages of heavy metals in such regional background soils are inevitable to be different from those in the background sediments of a lake.

Among the three provinces, the largest difference in metal contents between regional background soils and sediments is found in the two lakes in Inner Mongolia (Table 2; Figure 6). This corresponded well with the fact that Inner Mongolia (1.183 million km^2^) has the largest area among the three studied provinces. There are twelve main soil types and seven parent rock types in the province [43]. The second-largest difference is found in lake KL in Hebei Province. Hebei Province (188.8 thousand km^2^) has the second-largest area among the three provinces. It contains three main soil types: brown soil, tidal soil, and brown soil [44]. The smallest difference is found in the two lakes in Shanxi Province. The province is also the smallest among the three provinces, with an area of 156.7 thousand km^2^.

Among the nine metals, Cd and Hg often have the largest uncertainties in pollution and risk assessments when using regional background soils instead of background sediments in these lakes, while other metals show relatively minor uncertainties. For Cd and Hg, their pollution and risk assessment values calculated using background soils are usually one- to several-fold different from those using background sediments in the five lakes. Usually, the assessment values for Cd using background soils are higher than those using sediments, whereas those for Hg are lower. For example, the EF, PI, and RI values of Cd in the two lakes in Inner Mongolia assessed using background soils are nearly three times higher than those using sediments (Figure 3). The large differences in Cd and Hg are related to the fact that they have the lowest contents in background soil and sediment and obviously higher natural variabilities than other metals (Table 2). Standard deviations (SD) of Hg in background soils in all three provinces exceed the average values, and SDs of Cd in background soils in Inner Mongolia also exceed the average. Even in lakes of MY and GH in Shanxi Province, which are only ~5 km apart, the Hg concentration in MY background sediments (39.4 ± 1.8 ng/g) is twice that in GH (19.1 ± 2.2 ng/g). In contrast, the natural variability and SDs of other metals, which are much smaller than their average values, are relatively minor.

### 3.4. Pollution and Risks of the Nine Metals in the Lakes

From the results (NPI-sediment and RI-sediment) in Figure 5c,d, it can be seen that KL has obviously higher values of both NPI and RI compared to other lakes. Its NPI is as high as 4.2, suggesting a highly polluted level of heavy metals in its sediments. Its RI reaches 321, suggesting a considerable ecological risk level. For NPI in the other four lakes, GH and DL have values between 2 and 3, suggesting a moderately polluted level, while MY and ZS are between 1 and 2, suggesting a slightly polluted level. Although the four lakes show different pollution levels, their ecological risks are similar, with RI values between 162 and 232 suggesting a moderate ecological risk level. The pollution and ecological risk status/difference of heavy metals between these lakes correspond well with the fact that lake KL received more anthropogenic pollutant emissions than other lakes as it is situated close to (~5 km) a small county (Guyuan) [25]. In contrast, the other four lakes (i.e., GH, MY, DL, and ZS) are situated relatively far from urban/industrial areas, and thus they receive fewer anthropogenic pollutant emissions [23,25].

**Figure 7:**
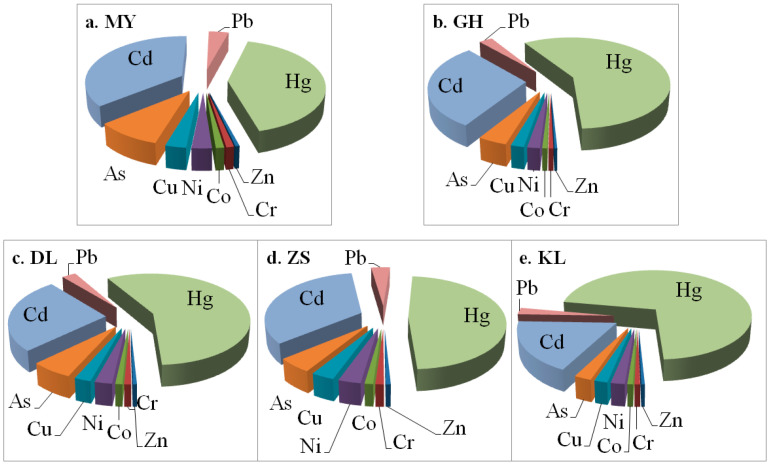
Contributions of the nine heavy metals to the potential ecological risks (RI) of the five lakes’ present sediments.

Among the nine metals, Hg is often the most polluted in these lakes. It usually shows moderately to highly polluted levels with considerable to high ecological risks. In these lakes, Hg contributes 42–71% of the potential ecological risks of all metals (Figure 7). The second is Cd. It shows moderate ecological risks in all five lakes, with slightly to moderately polluted levels. In these lakes, Cd contributes 18–36% of the potential ecological risks of all metals (Figure 7). Together, the total contribution of Hg and Cd is as high as 78–89% of the potential ecological risks of all metals (Figure 7). In contrast, the other seven metals (As, Co, Cr, Cu, Ni, Pb, and Zn) are basically unpolluted or slightly polluted with low ecological risks, implying they were mainly derived from natural sources.

Although Pb is a polluted metal in the global environment that has attracted special attention in recent decades [45,46,47], in all five lakes it is unpolluted and has very low ecological risks. This is likely due to the fact that these lakes are relatively remote and receive few anthropogenic Pb emissions derived mainly from urban/industrial areas.

The above environmental assessments suggest that the lakes received a large amount of heavy metals via regional atmospheric transport and deposition, although they are situated far from urban/industrial areas and were affected by limited human activities around them. This fits well with the previous conclusion that these lakes mainly received pollutants via regional atmospheric transport and deposition [23,25,27,29,42]. Hence, the different risks between metals were mainly related to atmospheric heavy metal emissions from anthropogenic sources in the region. This indicates the importance of controlling atmospheric anthropogenic Hg and Cd emissions in this and similar areas in China and guiding environmental management in rural and remote lakes as well as other water bodies.

## 4. Environmental Significance and Conclusions

In a comparative study of heavy metal assessments in five lakes’ sediments in North China, we found that there are large uncertainties in assessing heavy metal pollution and ecological risks using regional background soils instead of background sediments. Generally, the largest uncertainties are usually found in Hg and Cd, while uncertainties in other metals (Zn, Cr, Co, Ni, Cu, As, and Pb) are minor. This is caused by large differences in Hg and Cd contents between regional background soils and these lakes’ background sediments. Therefore, in the future, it will be necessary to investigate/obtain real background values of heavy metals in sediments before assessing metal pollution and ecological risks in Chinese lakes. Only in this way can we obtain reliable assessment results and understand the real pollution and ecological risks of heavy metals in lakes, which are helpful for establishing appropriate environmental management countermeasures.

In these lakes, the assessment results suggest moderate to high polluted levels of the nine heavy metals in most lakes’ present sediments and moderate to considerable ecological risks. Among the nine metals, Hg and Cd are the major (78–89%) contributors to the ecological risks in the lakes, while the other seven metals all have low ecological risks. The different risks between metals are mainly related to atmospheric heavy metal emissions from anthropogenic sources in the region, implying the importance of controlling atmospheric anthropogenic Hg and Cd emissions in this and similar areas in China.

Besides the five studied lakes, Hg and Cd are usually also heavily polluted metals in other Chinese lakes. However, in many previous investigations, they only assessed the pollution and risks of part of the heavy metals in lake sediments, not including Hg and/or Cd. Such assessments would significantly underestimate the pollution and environmental risks of heavy metals in lake sediments. In future studies, investigating heavy metals, including Hg and Cd, is crucial to obtaining reliable pollution and risk assessment results for toxic metals in lakes in China.

## Figures and Tables

**Table 1:** Details of the five lakes of GH, MY, DL, ZS, and KL in North China.

Province	Lake	Lake Location	Elevation (m)	Lake Area (km^2^)	Maximum Water Depth (m)
Shanxi	GH	38°54.3′–38°54.7′ N 112°14.3′–112°14.6′ E	1860	0.21	~10
	MY	38°51.9′–38°52.3′ N 112°12.3′–112°13.0′ E	1776	0.45	~9
Inner Mongolia	DL	43°13′–43°23′ N 116°29′–116°45′ E	1222	238	11
	ZS	43°41.0′–43°41.7′ N 116°53.1′–116°54.4′ E	1299	1.6	~2
Hebei	KL	41°42′0″–41°43′48″ N 115°41′42″–115°44′17″ E	1375	4.0	~6

**Table 2:** Statistical values of heavy metal concentrations in background and present sediments in the lakes and in regional background soils.

Province	Lake	Item	Ti	Zn	Cr	Co	Ni	Cu	As	Cd	Pb	Hg
						mg/kg						ng/g
**Shanxi**	**MY**	Average in 2010s	4290	97.2	66.2	12.4	29.4	22.9	13.4	0.25	24.0	66.4
		*SD*	*75*	*0.1*	*1.2*	*0.1*	*0.3*	*0.5*	*0.04*	*0.004*	*0.5*	
		Background	4607	97.6	71.5	12.9	31.5	23.2	8.7	0.13	19.1	39.4
		*SD*	*68*	*7.1*	*3.8*	*0.3*	*1.6*	*1.4*	*0.5*	*0.01*	*0.7*	*1.8*
		Max	4739	106.9	76.9	13.8	33.9	26.2	13.4	0.28	25.7	66.4
		Min	4236	89.0	65.4	12.3	29.1	21.5	8.3	0.12	18.2	35.8
	**GH**	Average in 2010s	3325	86.0	54.6	10.5	26.4	21.0	10.5	0.29	23.6	64.5
		*SD*	*59*	*3.6*	*0.6*	*0.1*	*0.3*	*0.5*	*0.4*	*0.02*	*0.3*	
		Background	3672	87.4	63.7	12.7	31.0	23.0	10.6	0.13	19.6	19.1
		*SD*	*21*	*0.6*	*0.4*	*0.2*	*0.3*	*0.3*	*0.1*	*0.004*	*0.2*	*2.2*
		Max	3833	91.7	64.0	12.9	31.4	23.2	12.5	0.31	24.8	75.6
		Min	3284	72.1	53.0	10.2	25.9	20.2	9.3	0.12	17.4	17.1
	**Soil**	Background	3800	71.9	61.5	10.8	33.7	28.3	10.4	0.13	18.1	24.0
		*SD*	*340*	*45.0*	*19.8*	*2.8*	*11.0*	*9.3*	*2.6*	*0.06*	*11.2*	*31.1*
**Inner Mongolia**	**DL**	Average in 2010s	2916	68.1	52.8	10.8	29.4	20.8	14.7	0.27	19.4	38.4
		*SD*	*90*	*0.7*	*0.3*	*0.2*	*0.2*	*0.2*	*0.4*	*0.005*	*0.4*	*2.8*
		Background	2967	56.2	50.8	10.4	27.6	20.2	11.2	0.15	16.7	12.5
		*SD*	*41*	*1.0*	*0.2*	*0.1*	*0.1*	*0.3*	*0.3*	*0.01*	*0.1*	*1.2*
		Max	3057	68.7	54.5	11.4	30.7	24.4	15.6	0.28	20.1	43.7
		Min	2813	55.6	50.5	10.3	27.5	20.0	10.9	0.14	16.6	11.0
	**ZS**	Average in 2010s	2685	62.1	41.6	8.0	22.9	17.4	11.8	0.25	16.9	50.0
		*SD*	*155*	*2.7*	*2.1*	*0.7*	*1.3*	*1.2*	*1.6*	*0.01*	*1.0*	*2.1*
		Background	2639	45.6	35.3	7.2	18.7	13.8	12.2	0.12	12.8	22.3
		*SD*	*112*	*0.4*	*1.0*	*0.1*	*0.3*	*0.002*	*0.9*	*0.005*	*0.1*	*1.4*
		Max	2888	66.4	45.9	9.0	25.2	19.7	14.6	0.27	18.5	52.4
		Min	2400	45.3	34.2	7.1	18.5	13.8	9.2	0.11	12.7	20.0
	**Soil**	Background	3200	56.7	38.0	10.1	19.0	13.5	7.5	0.05	16.8	34.0
		*SD*	*1030*	*40.2*	*23.9*	*6.0*	*13.1*	*8.5*	*6.2*	*0.06*	*9.8*	*46.1*
**Hebei**	**KL**	Average in 2010s	2958	87.5	58.5	12.1	34.2	26.5	13.0	0.28	22.5	72.0
		*SD*	*23*	*1.5*	*1.4*	*0.2*	*0.6*	*0.4*	*0.5*	*0.01*	*0.3*	*14.3*
		Background	2640	59.8	46.4	10.0	26.7	21.5	16.5	0.14	15.3	12.6
		*SD*	*116*	*2.0*	*0.7*	*0.5*	*0.8*	*1.2*	*1.3*	*0.01*	*0.7*	*0.4*
		Max	3274	89.5	60.3	12.5	34.9	27.0	18.3	0.28	22.8	87.3
		Min	2414	57.4	45.3	9.4	25.4	20.1	11.4	0.13	14.2	12.1
	**Soil**	Background	3600	78.4	72.6	16.7	34.1	23.0	14.2	0.10	25.1	22.0
		*SD*	*720*	*32.2*	*29.8*	*8.0*	*17.3*	*6.9*	*5.1*	*0.08*	*12.9*	*23.8*

## Data Availability

All data generated or analyzed during this study are included in this published article.
